# Association of a *TNIP1* Polymorphism with Vogt-Koyanagi-Harada Syndrome but Not with Ocular Behcet’s Disease in Han Chinese

**DOI:** 10.1371/journal.pone.0095573

**Published:** 2014-05-02

**Authors:** Yanyun Shi, Yading Jia, Shengping Hou, Jing Fang, Yan Zhou, Aize Kijlstra, Peizeng Yang

**Affiliations:** 1 The First Affiliated Hospital of Chongqing Medical University, Chongqing, China; 2 Shanxi Eye Hospital, Taiyuan, Shanxi, China; 3 Chongqing Eye Institute, Chongqing, China; 4 Chongqing Key Laboratory of Ophthalmology, Chongqing, China; 5 University Eye Clinic Maastricht, Maastricht, Limburg, The Netherlands; University of Iowa, United States of America

## Abstract

**Objectives:**

The aim of the study was to investigate the association of TNFα-induced protein 3 interacting with protein 1 (*TNIP1*) gene polymorphisms with Vogt–Koyanagi–Harada (VKH) syndrome and Behcet’s disease (BD) in a Han Chinese population.

**Methods:**

A total of 656 BD patients, 961 VKH syndrome patients and 1534 healthy controls were included in this two-stage case control study. Seven SNPs, including rs17728338, rs7708392, rs10036748, rs3762999, rs999556, rs4958881 and rs3792783, belonging to *TNIP1* were genotyped and analyzed by the polymerase chain reaction-restriction fragment length polymorphism (PCR-RFLP) method. The data were analyzed by using the χ2 or Fisher's exact test and corrected for multiple comparisons by the Bonferroni method.

**Results:**

A significantly increased frequency of the GG genotype and a decreased frequency of the AG genotype of rs17728338 were found in VKH patients (Pc = 0.038 OR = 1.934, 95% CI  = 1.438∼2.601). No significant difference was noted in allele or genotype frequencies of rs7708392, rs10036748, rs3762999, rs999556, rs4958881 and rs3792783, between VKH patients and healthy controls (Pc>0.05). No significant difference was noted in allele or genotype frequencies of the tested 7 SNPs between BD patients and healthy controls. Analysis of extraocular clinical findings, did not reveal an association of the *TNIP1* gene polymorphisms with BD or VKH syndrome subgroups.

**Conclusion:**

A *TNIP1* polymorphism may be a risk factor for VKH syndrome in Han Chinese.

## Introduction

TNFα-induced protein 3 interacting with protein 1(*TNIP1*), located on chromosome 5q32-q33.1, encodes the A20 binding inhibitor of the NF-κB1 (ABIN1) protein. *TNIP1* is an important regulator of NF-κB activity, playing an important role in maintaining homeostasis of the immune system [1]. Recently, genome-wide association and replication analysis studies have shown that genes in the NF-κB pathway such as TNFα-induced protein 3 (*TNFAIP3*) and *TNIP1* are associated with several autoimmune diseases including systemic lupus erythematosus (SLE) [2]–[5], psoriasis [6]–[7], psoriatic arthritis (PsA) [8], systemic sclerosis (SSc), and rheumatoid arthritis (RA) [9]–[10].

Vogt-Koyanagi-Harada (VKH) syndrome and Behçet’s disease (BD),manifesting as bilateral panuveitis,are two of the most common uveitis entities encountered in China [11]. Numerous studies have indicated that intrinsic factors play an important role in the development of these diseases. BD and VKH syndrome present a familial aggregation and a geographic distribution. BD is particularly common in populations of the Far East and the Mediterranean basin along the ancient silk route and VKH syndrome is mostly observed in Asians, Amerindians and Hispanics [12]. Human leukocyte antigen (HLA) class genes have been shown to be strongly associated with BD and VKH syndrome in populations with different ethnic backgrounds. HLA-DRB1,HLA-DR4/ DRw53 and HLA-DR1 are connected with the susceptibility to VKH syndrome in Japanese (90% of VKH patients have them) and the same results were found in Chinese, Indian, Korean, Mexican, and Hispanic patients [13]–[16]. HLA-B51/B5 is the most prominent immunogenetic susceptibility factor for BD in multiple ethnic groups [17].

However, HLA genes only account for part of the genetic-risk effect for VKH syndrome or Behcet’s disease, highlighting the fact that much of the heritable basis for these diseases remains unknown and implicating a possible role for non-HLA genes. Increasing evidence indicates that autoimmune diseases such as SLE, RA and Crohn’s disease share common risk genes with Behcet’s disease and VKH, including genes such as interleukin-23 receptor(*IL23R*) [18]–[20], protein tyrosine phosphatase non-receptor type 22 (*PTPN22*) [21]–[23] and signal transducer and activator of transcription 4 (*STAT4*) [24]–[25].

The aim of the present study was to investigate the association of *TNIP1* gene variants with the risk for BD and VKH syndrome, which to our knowledge, has not yet been reported.

## Materials and Methods

### Patients

A total of 656 BD patients, 961VKH syndrome patients and 1534 healthy controls were included in this two-stage study. All patients were recruited from the First Affiliated Hospital of Chongqing Medical University (Chongqing, China) or the Zhongshan Ophthalmic Center of Sun Yat-sen University (Guangzhou, China) and fulfilled the VKH syndrome and BD disease international criteria respectively [26]–[27]. All patients and controls belong to the Chinese Han population and were matched according to age and geographic area.

In this two-stage study, 377 BD, 374 VKH syndrome patients and 480 healthy controls were used to find out the susceptible SNPs (Pc<0.05) in the first stage study. In the second stage study, we added another 279 BD and/or 587 VKH syndrome patients and 1054 controls to replicate the associated SNPs indentified in the first stage study. Clinical findings of BD and VKH syndrome patients are presented in Table 1 and Table 2. This study was approved by the Ethics Committee of the First Affiliated Hospital of Chongqing Medical University. Written informed consent was obtained from all the subjects after explaining the purpose of the study. All procedures were carried out in compliance with the principles of the Declaration of Helsinki.

**Table 1:** Clinical features of BD patients used for the first and second stage study.

Clinical features	BD patients in the first stage Total(377)	%	BD patients in the second stage Total(279)	%
Age at onset (years±SD)	33.6±9.0		33.7±8.3	
Male	217	57.6	218	78.1
Female	160	42.4	61	21.9
Uveitis	377	100	279	100
Oral ulcer	355	94.2	266	95.3
Gentle ulcer	219	58.1	160	57.3
Hypopyon	95	25.2	75	26.9
Skin lisions	269	71.3	212	76.0
Positive pathergy test	104	27.6	32	11.5
Arthritis	57	15.1	25	9.0

**Table 2:** Clinical features of VKH syndrome patients used for the first and second stage study.

Clinical features	VKH patients in the first stage Total(374)	%	VKH patients in the second stage Total(587)	%
Age at onset (years±SD)	37.7±13.3		40.8±14.2	
Male	206	55.1	312	53.2
Female	168	44.9	275	46.9
Neck stiffness	34	9.1	71	12.1
Alopecia	139	37.2	246	41.9
Poliosis	124	33.2	233	39.7
Vitiligo	63	16.9	114	19.4
Dysacusia	82	21.9	217	37.0
Tinnitus	149	39.8	282	48.0
Scalp hypersensitivity	55	14.7	90	15.3

### SNP selection

SNP selection was based on published data. Seven SNPs of *TNIP1*, including rs17728338, rs7708392, rs10036748, rs3762999, rs999556, rs4958881 and rs3792783, were selected. These 7 SNPs have been proven to be associated with autoimmune diseases including SLE, psoriasis, psoriatic arthritis (PsA) and systemic sclerosis(SSc) [2]–[10]. The frequencies of these seven SNPs are higher than 0.10 in Chinese Han according to the dbSNP (http://www.ncbi.nlm.nih.gov/SNP/).

### Genotyping

Peripheral blood samples were collected in ethylenediamine tetraacetic acid (EDTA) anti-coagulated tubes and kept at −80°C until used. Genomic DNA was extracted by the QIAamp DNA Blood Mini Kit (Qiagen, Hilden, Germany) according to the manufacturer’s instructions. The target *TNIP1* gene sequences were amplified by polymerase chain reaction (PCR) and the 7 SNPs were subsequently genotyped and analyzed by polymerase chain reaction-restriction fragment length polymorphism (PCR-RFLP) analysis using primers,enzymes and conditions as shown in Table 3. The primers were designed using Primer Premier 5.0 software (Premier Biosoft International, Palo Alto, CA).

**Table 3:** Primer sequences and restriction enzymes used for RFLP analysis of the *TNIP1* gene.

SNP	Forward primer sequence	Reverse primer sequence	Tm(°C)	Enzyme
rs17728338	TTCAGAACAGTGGCTACTCTCCTCCTC	CATTCGGGAGCCTTTTGCCA	66	NCOI
rs7708392	TTTCCCAATGCTGCTAAGGA	TGGAACCTGGGTCTCTTCTG	60	Hpy188I
rs10036748	CACGTATGAGAAAAATAAAATAGTCA	GATCAAGTCTCAGCTCAAATGT	56	Hsp92II
rs3762999	TAGGCAAGTTTTGGTCCTCAGCATC	GGCAATCCGCCTTTATTCATCCT	66	BSEGI
rs999556	AGGCTTCCTATCCACCACCTA	AGACACTGCTCCTTCCTCTCC	60	BCNI
rs4958881	GCTATAAGAAGCCCTTGAACCA	GTTCCCTCTTGGTGGTGTATGT	60	Hin1I
rs3792783	CCAGAGAGTTGCCACAGGAAG	CTGTGTAGGTGCTCAAGGACG	61	XcmI

Each PCR reaction was performed in 6 µl containing 3 µl Premix Taq (Promega, Madison, USA), 2 pmoles primers and 0.1 µg of genomic DNA. The PCR conditions were as follow: initial denaturation at 95°C for 5 minutes followed by 36 cycles of denaturation at 94°C for 30 seconds, annealing at 56–58°C (56°C for rs17728338, rs7708392, rs10036748 and rs3762999, 58°C for rs999556, rs4958881 and rs3792783) for 30 seconds, extension at 72°C for 30 seconds, and a final extension at 72°C for 5 minutes. PCR products were digested respectively with 2 U restriction enzymes (Table 3) in 8 µl reaction volume overnight. Digestion products were visualized on a 4%–5% agarose gel and stained with GoldView (SBS Genetech, Beijing, China).

Randomly selected subjects (10% of all samples) were directly sequenced (Biomed, Beijing, China) to double check the validation of the PCR-RFLP results.

### Statistical analysis

Data analysis was performed using an SPSS statistical package (version 17.0, SPSS Inc., Chicago, IL, USA). Genotype frequencies were calculated by direct counting. Chi-square analysis was used to test for deviation of genotype frequencies from Hardy–Weinberg equilibrium. The frequency of alleles and genotypes between patients and controls were compared using χ^2^ test or Fisher’s exact test and the probability of an association was corrected with the Bonferroni method. *P* Bonferroni correction values of <0.05 were considered significant. Odds ratios (ORs) and 95% confidence intervals (95% CI) were also assessed.

## Results

Genotype frequencies of tested seven SNPs in this study did not deviate from Hardy-Weinberg equilibrium in the case and control group (*P*>0.05). In the first stage study, 377 BD, 374 VKH syndrome patients and 480 healthy controls were randomly chosen from the whole patient and healthy control cohort to find out the susceptible SNPs (*Pc*<0.05). The results showed that the frequencies of the GG genotype and G allele of rs17728338 were significantly increased in VKH patients (Bonferroni *Pc* = 0.019, OR = 2.265, 95% CI  = 1.400-3.363; *Pc* = 0.014, OR = 2.170, 95% CI = 1.357-3.471; respectively). No significant difference was noted in allele or genotype frequencies of rs7708392, rs10036748, rs3762999, rs999556, rs4958881 and rs3792783, between VKH patients and healthy controls (Pc>0.05). No significant difference was noted in allele or genotype frequencies of all tested 7 SNPs between BD patients and healthy controls. (Table 4)

**Table 4:** Frequencies of alleles and genotypes of *TNIP1* polymorphisms in BD, VKH patients and controls in the first stage study.

SNPs	Genotype/allele	VKH(n = 374)	BD (n = 377)	Controls (n = 480)	P^a^ / Pc^a^	OR(95%CI)	P^b^ /Pc^b^	OR(95%CI)
rs17728338	GG	349(93.3)	342(90.7)	413(86.0)	0.001/0.019	2.265(1.400–3.363)	0.036/ NS	1.585(1.028–2.445)
	AG	25(6.7)	35(9.3)	67(14.0)				
	G	723(96.7)	719(95.4)	893(93.0)	0.001/0.014	2.170(1.357–3.471)	0.115/ NS	2.170(1.357–3.471)
	A	25(3.3)	35(4.6)	67(7.0)				
rs7708392	CC	186 (49.7)	177(46.9)	244(50.8)	0.750/ NS	0.957(0.730–1.254)	0.259/ NS	0.856(0.653–1.121)
	CG	152 (40.6)	168(44.6)	187(39.0)				
	GG	36(9.6)	32(8.5)	49(10.2)				
	C	524(70.1)	522(69.2)	675(70.3)	0.908/ NS	0.988(0.801–1.217)	0.628/ NS	0.950(0.772–1.169)
	G	224(29.9)	232(30.8)	285(29.7)				
rs10036748	TT	221(59.1)	223(59.2)	277(57.7)	0.684/ NS	1.059(0.805–1.393)	0.987/ NS	0.998(0.746–1.334)
	CT	138(36.9)	138(36.6)	183(38.1)				
	CC	15(4.0)	16(4.2)	20(4.2)				
	T	580(77.5)	584(77.5)	737(76.8)	0.707/ NS	1.405(0.832–1.312)	0.739/ NS	1.039(0.828–1.305)
	C	168(22.5)	170(22.5)	223(23.2)				
rs3762999	AA	223(59.6)	218(57.8)	295(61.5)	0.586/ NS	0.926(0.702–1.221)	0.281/ NS	0.860(0.653–1.132)
	AG	136(36.4)	142(37.7)	171(35.6)				
	GG	15(4.0)	17(4.5)	14(2.9)				
	A	582(77.8)	578(76.7)	761(79.3)	0.464/ NS	0.917(0.727–1.157)	0.194/ NS	0.859(0.682–1.081)
	G	166(22.2)	176(23.3)	199(20.7)				
rs999556	AA	11(2.9)	13(3.4)	15(3.1)	0.877/ NS	0.939(0.426–2.070)	0.693/ NS	0.848(0.375–1.919)
	AG	132(35.8)	142(37.7)	171(35.6)				
	GG	231(61.8)	222(58.9)	294(61.3)				
	A	154(20.6)	168(22.3)	201(20.9)	0.860/ NS	0.979(0.773–1.239)	0.502/ NS	1.083(0.859–1.365)
	G	594(79.4)	586(77.7)	759(79.1)				
rs4958881	CT	40(10.6)	37(9.8)	71(14.8)	0.077/ NS	0.690(0.456–1.043)	0.029/ NS	0.627(0.411–0.957)
	TT	334(89.4)	340(90.2)	409(85.2)				
	C	40(5.3)	37(4.9)	71(7.4)	0.088/ NS	0.707(0.474–1.055)	0.035/ NS	0.646(0.429–0.973)
	T	708(94.7)	717(95.1)	889(93.6)				
rs3792783	CC	11(2.9)	12(3.2)	15(3.1)	0.877/ NS	0.939(0.426–2.070)	0.962/ NS	1.019(0.471–2.204)
	CT	125(33.4)	120(31.8)	168(35.0)				
	TT	238(63.6)	245(65.0)	297(61.9)				
	C	147(19.7)	144(19.1)	198(20.6)	0.619/ NS	0.941(0.741–1.195)	0.432/ NS	0.908(0.715–1.154)
	T	601(80.3)	610(80.9)	762(79.4)				

CI, confidence intervals; OR, odds ratios; NS, not significant; *P*c value, the Bonferroni correction *P* values. *P*c^a^ value,the Bonferroni correction *P* values for VKH syndrome; *P*c^b^ value the Bonferroni correction *P* values for BD.

In the second stage study, we replicated the association of the rs17728338 polymorphism using another set of 587 VKH patients and 1054 controls. Genotypes distribution and allele frequencies are shown in Table 5. The results showed a significantly increased frequency of the GG genotype and a decreased frequency of the AG genotype in VKH patients (*Pc* = 0.038; *Pc* = 0.038). The combined results also showed a significant association of SNP rs17728338 with VKH syndrome (*Pc* = 1.83×10^−4^, OR = 1.934, 95% CI  = 1.438–2.601).

**Table 5:** Frequencies of alleles and genotypes of *TNIP1* polymorphisms in VKH patients and controls in the second stage and combined results.

Genotype/allele	VKH (n = 587)	Controls (n = 1054)	*P*value(OR,95%CI)	*P*c value	*P* ^combined^ value(OR, 95%CI)	*P*c^combined^ value
GG	548(93.3%)	935(88.9%)	0.002(OR = 1.788, 95% CI = 1.227-2.606)	0.038	9.67×10^−6^(OR = 1.934 95% CI = 1.438-2.601)	1.83×10^−4^
AG	39(6.7%)	119(11.1%)				
G	1135(96.7%)	1993(94.5%)	0.003(OR = 1.765, 95% CI = 1.221-2.553)	0.042	1.74×10^−5^(OR = 1.871, 95% CI = 1.400-2.500)	2.44×10^−4^
A	39(3.3%)	119(5.5%)				

Subdivision of the patients according to the extraocular clinical findings, did not reveal an association of the tested seven SNPs with BD or VKH syndrome subgroups.

## Discussion

In this study we show an association between a *TNIP1* rs17728338 gene polymorphism with VKH syndrome but not with BD. We found that the frequence of the AG genotype of rs17728338 was decreased in VKH patients, which suggests that it may play a protective role in the development of VKH syndrome in the Chinese Han population.

As a chronic multisystemic relapsing inflammatory disorder, VKH syndrome is characterized as a bilateral granulomatous panuveitis accompanied by meningitis, vitiligo, alopecia, poliosis, tinnitus and hearing loss [28]–[29]. BD is characterized as a relapsing uveitis accompanied by recurrent oral aphthous ulcers, genital ulcerations with mucocutaneous, articular, neurologic, urogenital and vascular manifestations [30]. Although clinical features of both diseases have been described comprehensively, their precise etiology and pathogenesis are still unclear. Other autoimmune diseases such as SLE, RA and psoriasis, which had been found with association with *TNIP1* polymorphisms, may share autoimmune features and mechanism with VKH syndrome and Behçet’s disease (BD) [31].

*TNIP1* is a newly discovered gene influencing susceptibility to multiple immune-related diseases [2]–[10]. One of the important functions of *TNIP1* is to regulate NF-κB pathways. NF-κB pathways may promote the expression of genes and secretion of cytokines by human uveal melanocytes, which are considered antigen-presenting cells in human Vogt-Koyanagi-Harada disease [32]. *TNIP1* can inhibit signaling pathways of various transmembrane receptors, such as the TNFα-receptor, epidermal growth factor receptor (EGF-R), toll-like receptors (TLR), nuclear receptors peroxisome proliferator-activated receptors (PPARs) and retinoic acid receptors (RARs) [33]–[35]. These receptors play key roles in regulating inflammation and inflammatory diseases.

A polymorphism of rs17728338 near *TNIP1* was first reported to be associated with psoriasis in Americans from European ancestry and subsequently confirmed in other Caucasian populations with PsA [2] [22]. In our study,we genotyped 7 SNPs of *TNIP1*. One of these SNPs, rs17728338, was observed to be associated with VKH syndrome, whereby the frequency of the A allele was shown to be decreased in VKH syndrome patients. Whether the observed rs17728338 polymorphism has implications concerning the biological function of *TNIP1* is not yet known as deserves further study. The other 6 SNPs were not associated with VKH although earlier studies showed that there was an association with autoimmune diseases such as SLE, RA and psoriasis. This suggests that the pathogenesis of VKH is different from these immune mediated diseases. This is not surprising since VKH is unique due to the fact that it is an autoimmune disease directed against melanocyte antigens.

We also investigated the relationship between the clinical features of VKH syndrome patients and *TNIP1* polymorphisms. These clinical features include neck stiffness, tinnitus, alopecia, poliosis, dysacusia, scalp hypersensitivity, and vitiligo. No significant association could be detected between *TNIP1* gene polymorphisms and the clinical features of VKH syndrome, but a larger sample size may be needed to resolve this issue.

There are limitations in our study. First, it is not clear whether the observed *TNIP1* rs17728338 polymorphism has implications concerning the biological function of *TNIP1*. Second, we did not observe an association with *TNIP1* gene polymorphisms with BD and it is possible that other relevant polymorphisms of this gene are associated with this uveitis entity. Furthermore the association of the *TNIP1* gene association with VKH syndrome was only performed in a Chinese Han population and further studies are needed in other ethnic populations.
